# circRNF20 aggravates the malignancy of retinoblastoma depending on the regulation of miR-132-3p/PAX6 axis

**DOI:** 10.1515/med-2022-0483

**Published:** 2022-05-25

**Authors:** Dexiang An, Jing Yang, Linli Ma

**Affiliations:** Department of Ophthalmology, Lianyungang Maternal and Child Health Hospital, Lianyungang, Jiangsu Province, People’s Republic of China; Department of Pharmacy, Lianyungang Maternal and Child Health Hospital, Lianyungang, People’s Republic of China; Department of Ophthalmology, The Second People’s Hospital of Lianyungang, No. 41 Hailian Dong Road, Haizhou District, Lianyungang 222000, People’s Republic of China; Department of Ophthalmology, The Oncology Hospital of Lianyungang, No. 41 Hailian Dong Road, Haizhou District, Lianyungang 222000, Jiangsu Province, People’s Republic of China

**Keywords:** RB, circRNF20, miR-132-3p, PAX6

## Abstract

Circular RNAs (circRNAs) serve as essential players in diverse human cancers, including retinoblastoma (RB). In this study, the function of circRNA Ring Finger Protein 20 (circRNF20) in RB progression was investigated. Quantitative real-time polymerase chain reaction, western blot assay or immunohistochemistry assay was performed to determine the expression of circRNF20, miR-132-3p and Paired Box 6 (PAX6). Dual-luciferase reporter assay, RNA immunoprecipitation assay and RNA pull-down assay were utilized to verify the relationships among circRNF20, miR-132-3p and PAX6. *In vivo* experiment was done for circRNF20 function in tumor formation. It was found that ircRNF20 level was increased in RB tissues and linked to advanced tumor, nodes, metastases (TNM) stage and poor overall survival rate. Deficiency of circRNF20 suppressed cell proliferation, migration and invasion and induced apoptosis *in vitro*, as well as blocked tumor growth *in vivo*. circRNF20 directly targeted miR-132-3p and miR-132-3p overexpression inhibited RB cell progression. PAX6 was the target gene of miR-132-3p. Moreover, miR-132-3p inhibition or PAX6 overexpression reversed circRNF20 deficiency-mediated effects on RB cell malignant behaviors. In addition, exosomal circRNF20 was able to promote RB cell progression. Thus, we concluded that circRNF20 served as an oncogene in RB progression through the circRNF20/miR-132-3p/PAX6 pathway.

## 1 Introduction

Retinoblastoma (RB) is a common ocular malignancy in children [1,2]. RB has attracted extensive attention in the medical field because of its unique genetic law, multidirectional differentiation potential and high degeneration rate [3]. Early effective treatment can preserve some visual functions and prolong the survival of children [4]. However, if not diagnosed and treated on time and the optimal treatment time is missed, it will lead to poor prognosis in RB patients [5]. Therefore, it is urgent to clarify the basic mechanism of the development of RB, find useful biomarkers and explore novel targets for RB therapy.

Circular RNAs (circRNAs) represent a class of endogenous RNAs which are featured by closed-loop structures [6]. circRNAs can modulate the biological functions via the competitive endogenous RNA mechanism, that is, functioning as microRNA (miRNA) sponges, thereby regulating gene expression [7]. Recently, the contributions of circRNAs in cancer progression have been widely reported. For example, circ_0069094 played an oncogenic role in breast cancer development via altering miR-661 and HMGA1 expression [8]. Circ_0000629 decelerated the carcinogenesis of bladder cancer by the miR-1290/CDC73 pathway [9]. In RB, some circRNAs, such as circTET1 [10], circ_0000034 [11] and circ_0001649 [12], were reported to be involved in RB advancement. CircRNA Ring Finger Protein 20 (circRNF20, hsa_circ_0087784) served as a tumor accelerator in non-small-cell lung carcinoma (NSCLC) and breast cancer [13,14]. Nonetheless, the relationship between circRNF20 and RB development is not clear.

miRNAs are short noncoding RNAs that can influence gene expression by combining with the 3′-UTR of target mRNAs [15]. Increasing evidence has documented the involvement of miRNAs in RB. Li et al. reported that miR-218-5p blocked the malignancy of RB through altering NACC1 expression and AKT/mTOR pathway [16]. Zhang and Wu claimed that miR-378a-3p exerted an anticancer effect on RB by binding to FOXG1 [17]. As for miR-132-3p, Han et al. declared its inhibitory effect on RB progression [18]. However, the exact roles of miR-132-3p in RB still need further research.

Paired Box 6 (PAX6) belongs to the PAX gene family and is a regulator of retinal formation and the development of other eye tissues [19,20]. Furthermore, PAX6 was reported to serve as the target of some miRNAs to participate in RB development [21,22,23]. Even so, whether PAX6 can be targeted by miR-132-3p is unknown.

In this study, starbase (http://starbase.sysu.edu.cn/) analysis exhibited that miR-132-3p contained the binding sites of circRNF20 and PAX6. Thus, we explored the effects and relationships of circRNF20, miR-132-3p and PAX6 in RB development.

## 2 Materials and methods

### 2.1 Tissue and serum collection

A total of 52 RB tissues were collected from RB patients and 22 normal tissues were collected from patients with ophthalmorrhexis treated with enucleation at Lianyungang Maternal and Child Health Hospital. The serum samples were also collected from 38 RB patients and 20 normal patients. None of the patients received chemo- and radiotherapy before the surgery. The tumor and serum samples were saved at −80°C till use. The work was authorized by the Ethics Committee of Lianyungang Maternal and Child Health Hospital and written informed consents were offered by the participants.

### 2.2 Cell culture

RB cell line WERI-Rb-1 and normal cell line ARPE-19 were provided by Procell (Wuhan, China). RB cell line Y79 was acquired from BNBIO (Beijing, China). RB cell line SO-RB50 was purchased from Shanghai Qincheng Bio (Shanghai, China). These cells were grown at 37°C in DMEM (Procell) plus 10% FBS (Procell) and 1% penicillin/streptomycin (Procell) in an incubator containing 5% CO_2_.

### 2.3 Quantitative real-time polymerase chain reaction (qRT-PCR)

The RNAs were extracted via an Rneasy Mini kit (Qiagen, Inc., Valencia, CA, USA). Then, reverse transcription experiments were conducted on the RNAs through the usage of miRNA cDNA Synthesis reagent (Vazyme, Nanjing, China) or M-MLV Reverse Transcriptase reagent (Promega, Madison, WI, USA) in line with the protocols. Next, qRT-PCR was manipulated by using SYBR Green qPCR mix (Takara, Dalian, China). The 2^−ΔΔCt^ method was employed to compute the expression. GAPDH and U6 served as internal controls. The primers used are exhibited in Table 1.

**Table 1:** Primer sequences used for qRT-PCR

Name		Primers for PCR (5′–3′)
circRNF20	Forward	GAGCCGTGTCCCAGATTGT
	Reverse	TGCCGCTGATCCAACATTTC
RNF20	Forward	CCTCACTATTGATTGTCAACCGA
	Reverse	TCATCTTTACGCTCCTGATTGC
miR-132-3p	Forward	TGCGGTAACAGTCTACAGCCATG
	Reverse	CCAGTGCAGGGTCCGAGGT
PAX6	Forward	AGTGCCCGTCCATCTTTGC
	Reverse	CGCTTGGTATGTTATCGTTGGT
GAPDH	Forward	GACAGTCAGCCGCATCTTCT
	Reverse	GCGCCCAATACGACCAAATC
U6	Forward	CTCGCTTCGGCAGCACA
	Reverse	AACGCTTCACGAATTTGCGT

### 2.4 Actinomycin D assay and RNase R assay

To block transcription, Y79 and WERI-Rb-1 cells were treated with Actinomycin D (1 mg/mL; Sigma-Aldrich, St. Louis, MO, USA) for indicated times.

For RNase R treatment, total RNA was managed with RNase R (Epicenter, Madison, WI, USA) for 15 min at 37°C.

After the above treatment, circRNF20 and RNF20 mRNA levels were examined through qRT-PCR assay.

### 2.5 Cell transfection

To silence circRNF20, short hairpin RNAs against circRNF20 (sh-circRNF20#1, sh-circRNF20#2 or sh-circRNF20#3) were transfected into Y79 and WERI-Rb-1 cells and sh-NC served as a control. To overexpress or inhibit miR-132-3p, miR-132-3p mimics (miR-132-3p) and miR-132-3p inhibitors (anti-miR-132-3p) were used with miR-NC and anti-NC as controls. PAX6 overexpression vector was transfected into RB cells to elevate PAX6 expression with empty vector (vector) as a control. Lipofectamine 2000 (Invitrogen, Carlsbad, CA, USA) was applied for the transfection referring to protocols.

### 2.6 Cell counting kit-8 (CCK-8) assay

To assess cell viability, cells were plated into 96-well plates (5 × 10^3^ cells per well) and incubated with CCK-8 solution (Beyotime, Shanghai, China) at 48 h. After 2 h, the OD value (450 nm) in each well was examined by a microplate reader.

### 2.7 5-Ethynyl-2′-deoxyuridine (EdU) detection

By using an EdU assay kit (Beyotime), cell proliferation ability was examined. In short, cells were grown in 24-well plates (5 × 10^3^ cells per well) and then kept with EdU for 2 h. Next, the cells were fixed with 4% paraformaldehyde (Sigma-Aldrich) for 0.5 h and permeabilized with 0.5% Triton X-100 (Sigma-Aldrich) for 20 min. Afterward, the cells were washed with PBS and dyed with Apollo and DAPI. The EDU-positive cells were quantified under a fluorescence microscope.

### 2.8 Colony formation analysis

Y79 and WERI-Rb-1 cells were cultured in 6-well plates for about 12 days. Then, the colonies were interacted with 0.1% crystal violet (Sigma-Aldrich) for staining. The number of colonies was counted.

### 2.9 Flow cytometry analysis

The apoptosis of Y79 and WERI-Rb-1 cells was analyzed as per the instructions of an Annexin V-fluorescein isothiocyanate/propidium iodide Apoptosis Detection Kit (Beyotime) and estimated by FACScan^®^ flow cytometry (BD Biosciences, San Jose, CA, USA).

### 2.10 Wound-healing assay

The migration capacity of Y79 and WERI-Rb-1 cells was tested by this assay. In short, the cells were housed in 12-well plates and allowed to achieve 90% confluence. Next, the scratches were made using a pipette tip. The scratch width was observed at 0 and 24 h.

### 2.11 Transwell assay

The transwell chambers (BD Biosciences) pre-covered with Matrigel (BD Bioscience) were utilized for cell invasion analysis. In short, RB cell suspension (1 × 10^4^ cells per mL) was added into the top compartment and the complete culture medium was added into the lower compartment. The invaded cells were dyed with 0.1% crystal violet (Sigma-Aldrich) after 24 h and quantified under a microscope.

### 2.12 Western blot assay

The proteins were obtained by using RIPA (Beyotime) and then subjected to SDS-PAGE electrophoresis. Next, the samples were electroblotted onto PVDF membranes and blocked in 5% skim milk for 2 h. Thereafter, the membranes were kept overnight with primary antibodies against GAPDH (ab181602; Abcam), proliferating cell nuclear antigen (PCNA; ab18197; Abcam, Cambridge, MA, USA), cleaved-caspase 3 (ab2302; Abcam), E-cadherin (ab231303; Abcam), N-cadherin (ab207608; Abcam), vimentin (ab137321; Abcam) or PAX6 (ab195045; Abcam) and secondary antibody (ab205718; Abcam) for 2 h. The bands were exposed using an ECL kit (Beyotime).

### 2.13 Subcellular fraction analysis

PARIS Kit (Life Technologies, Austin, Texas, USA) was used to separate the cytoplasm and nucleus from Y79 and WERI-Rb-1 cells with GAPDH and U6 as the controls for cytoplasm and nucleus, respectively.

### 2.14 Dual-luciferase reporter assay

The fragments of wild type (WT) circRNF20 or PAX5 3′-UTR containing miR-132-3p binding sites and mutant (MUT) fragments lacking miR-132-3p binding sites were cloned and introduced into pmirGLO (Promega, Fitchburg, WI, USA). Next, the vectors and miR-132-3p/miR-NC were co-introduced into Y79 and WERI-Rb-1 cells. After 48 h, the luciferase signal was detected using a Dual-Luciferase Reporter Assay kit (Promega).

### 2.15 RNA immunoprecipitation (RIP) assay

The Magna RIP kit (Millipore, Billerica, MA, USA) was utilized for this experiment. In short, after lysed in RIP buffer, Y79 and WERI-Rb-1 cell extracts were incubated with anti-Ago2 (ab32381; Abcam) or anti-IgG (ab48386; Abcam) coupled magnetic beads. Next, the abundance of circRNF20 and miR-132-3p in the immunoprecipitated complexes was detected.

### 2.16 RNA pull-down assay

miR-132-3p biotin-labeled probe (Bio-miR-132-3p) or related control (Bio-miR-NC) were transfected into RB cells. Then, cell lysates were incubated with streptavidin-covered magnetic beads. The supernatants were collected and coprecipitated RNA was detected.

### 2.17 *In vivo* experiment

The BALB/c nude mice were obtained from Beijing Vital River Laboratory Animal Technology Co., Ltd. (Beijing, China) and divided into two groups (*n* = 5 mice/group). Sh-NC or sh-circRNF20#1 transfected Y79 cells were introduced into the mice. The volume of formed tumors was detected every 7 days and evaluated with the formula: volume = 0.5 × length × width^2^. On day 35, the mice were euthanized and tumors were removed and preserved at −80°C until further use. The *in vivo* study was approved by the Ethics Committee of Animal Research of Lianyungang Maternal and Child Health Hospital.

### 2.18 Immunohistochemistry (IHC) assay

After the tumor tissues were sectioned at 5 μm, IHC assay was utilized to examine the levels of ki-67 and PAX6, as previously reported [24]. The primary antibodies ki-67 (ab15580) and PAX6 (ab195045) and goat anti-rabbit HRP secondary antibody (ab205718) were obtained from Abcam.

### 2.19 Isolation and identification of exosomes

The isolation of exosomes was executed by using an ExoQuick precipitation kit (System Biosciences, Mountain View, CA, USA). In short, the samples were centrifuged at 3,000×*g* for 20 min. After filtration with a 0.22 mm PVDF filter, the samples were added with precipitant, mixed, shaked and then stored in the refrigerator at 4°C for 30 min. Thereafter, the samples were centrifuged at 1,500×*g* for 15 min. The supernatant was discarded and exosomes were suspended in PBS and placed at −80°C until use.

The exosome morphology was observed under transmission electron microscopy according to the manufacturers’ instructions.

To identify the exosomes, exosomal proteins HSP70 and TSG101 were measured. The related primary antibodies (ab2787; ab30871) were offered by Abcam.

### 2.20 Statistical analysis

All experiments were conducted triple times and exhibited as mean ± standard deviation. Data analysis was conducted using GraphPad Prism 7. The survival curve was obtained through Kaplan–Meier plot and estimated by log-rank test. Spearman’s correlation coefficient was performed to analyze the correlation between the circRNF20 level and miR-132-3p level. Student’s *t*-test or one-way analysis of variance was used for difference analysis. *P* < 0.001 was considered to be significantly different.

## 3 Results

### 3.1 CircRNF20 was upregulated in RB tissues and cells

**Figure 1:**
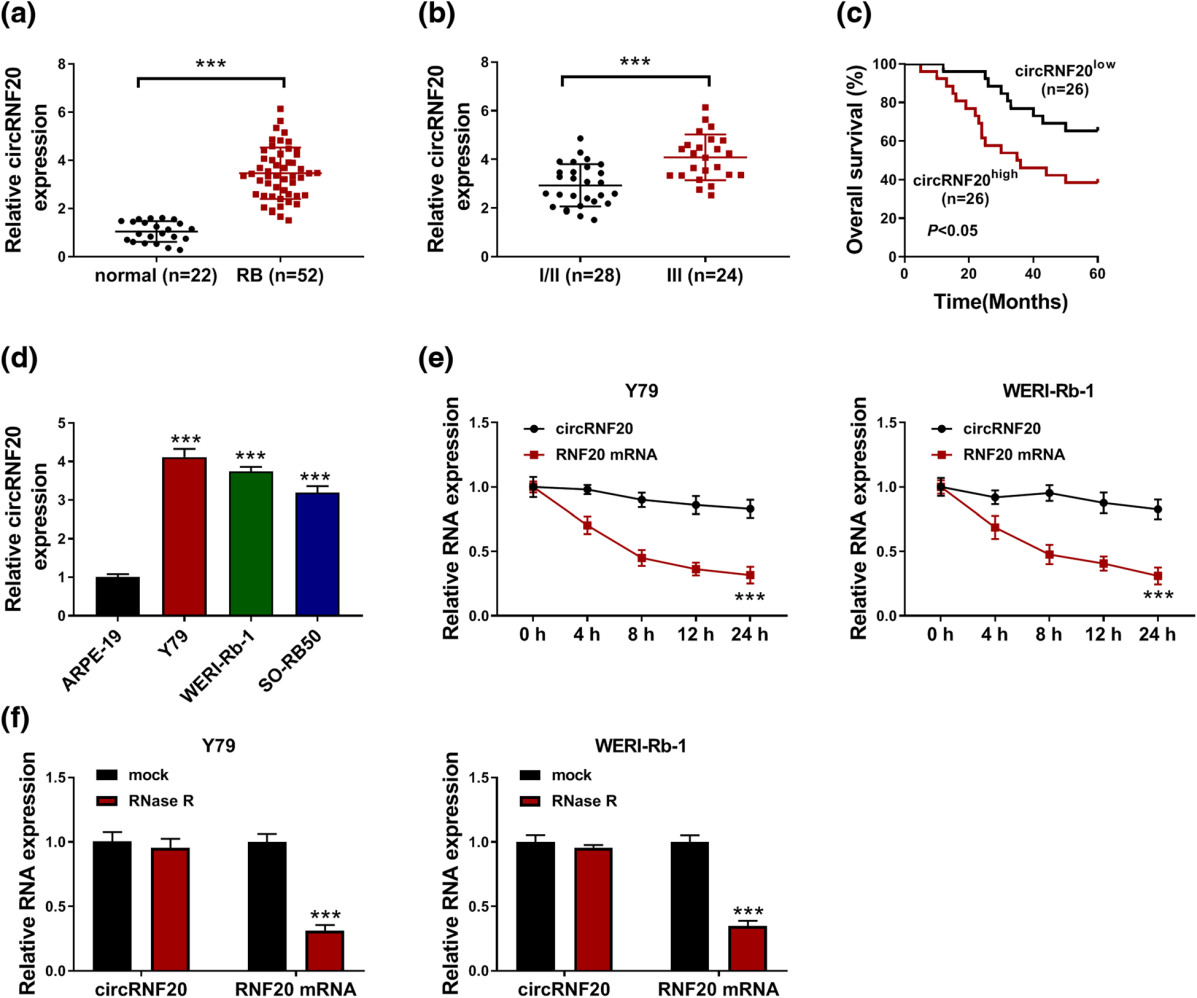
circRNF20 was increased in RB tissues and cells. (a) The expression of circRNF20 in RB tissues and normal tissues was detected by qRT-PCR assay. (b) The expression of circRNF20 in RB tissues at different TNM stages was detected by qRT-PCR assay. (c) The overall survival rate of RB patients in circRNF20^low^ and circRNF20^high^ groups was analyzed. (d) The level of circRNF20 in ARPE-19, Y79, WERI-Rb-1 and SO-RB50 cells was detected by qRT-PCR assay. (e) After Y79 and WERI-Rb-1 cells were treated with actinomycin D for indicated times, the levels of circRNF20 and RNF20 mRNA were detected by qRT-PCR assay. (f) The levels of circRNF20 and RNF20 mRNA in Y79 and WERI-Rb-1 cells treated with or without RNase R were detected by qRT-PCR assay. ***P < 0.001.

Initially, the expression level of circRNF20 in RB tissues (*n* = 52) and normal tissues (*n* = 22) was determined by qRT-PCR assay. The results showed that circRNF20 was markedly upregulated in RB tissues compared to normal tissues (Figure 1a). Compared to tumors at TNM I/II stages (*n* = 28), tumors at TNM III (*n* = 24) stages showed higher expression of circRNF20 expression (Figure 1b). Moreover, the RB patients were divided into two groups (circRNF20^low^ and circRNF20^high^) according to the median of circRNF20 expression in 52 RB tissues. Our results showed that the overall survival rate of RB patients in the circRNF20^high^ group was lower than patients in the circRNF20^low^ group (Figure 1c). qRT-PCR assay indicated that circRNF20 was markedly increased in RB cell lines (including Y79, WERI-Rb-1 and SO-RB50) compared to normal cell line (ARPE-19) (Figure 1d). Actinomycin D assay showed that the half-life of RNF20 was lower than circRNF20 (Figure 1e). RNase R assay indicated that circRNF20 was resistant to RNase R treatment, whereas RNF20 was markedly digested by RNase R (Figure 1f). These results indicated that circRNF20 was stable in RB cells and its abnormal expression might be involved in the progression of RB.

### 3.2 CircRNF20 knockdown suppressed RB cell proliferation, migration and invasion and promoted apoptosis

**Figure 2:**
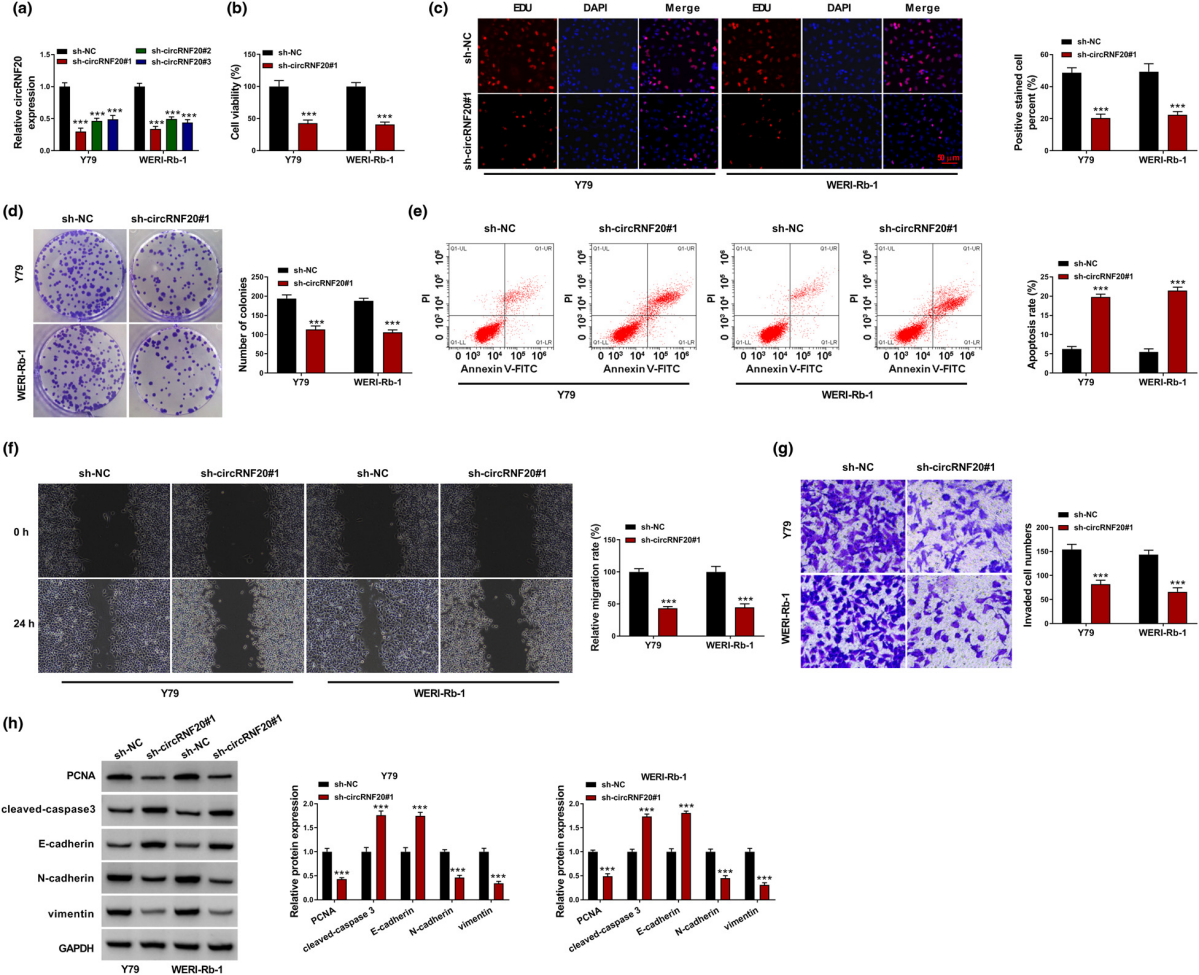
Effects of circRNF20 knockdown on RB cell proliferation, apoptosis, migration and invasion. (a) The expression of circRNF20 in Y79 and WERI-Rb-1 cells transfected with sh-NC, sh-circRNF20#1, sh-circRNF20#2 and sh-circRNF20#3 was detected by qRT-PCR assay. (b–h) Y79 and WERI-Rb-1 cells were transfected with sh-NC or sh-circRNF20#1. (b–d) The viability, proliferation and colony formation of Y79 and WERI-Rb-1 cells were assessed by CCK-8 assay, EDU assay and colony formation assay. (e) The apoptosis of Y79 and WERI-Rb-1 cells was analyzed by flow cytometry analysis. (f and g) The migration and invasion of Y79 and WERI-Rb-1 cells were evaluated by wound-healing assay and transwell assay, respectively. (h) The protein levels of PCNA, cleaved-caspase 3, E-cadherin, N-cadherin and vimentin in Y79 and WERI-Rb-1 cells were measured through western blot assay. ***P < 0.001.

To investigate the functions of circRNF20 in RB progression, Y79 and WERI-Rb-1 cells were transfected with sh-circRNF20#1, sh-circRNF20#2 or sh-circRNF20#3 to silence circRNF20 expression. qRT-PCR assay showed that sh-circRNF20#1, sh-circRNF20#2 or sh-circRNF20#1 transfection led to a notable reduction in circRNF20 expression in both Y79 and WERI-Rb-1 cells in comparison with sh-NC control groups (Figure 2a). As indicated by CCK-8 assay, the Y79 and WERI-Rb-1 cells with circRNF20 knockdown showed the inhibited cell viability compared to cells with sh-NC transfection (Figure 2b). EDU assay showed that circRNF20 knockdown repressed the proliferation ability of Y79 and WERI-Rb-1 cells (Figure 2c). The results of colony formation assay exhibited that circRNF20 deficiency restrained the colony formation ability of Y79 and WERI-Rb-1 cells compared to sh-NC control groups (Figure 2d). Flow cytometry analysis indicated that circRNF20 silencing promoted the apoptosis rate of Y79 and WERI-Rb-1 cells relative to sh-NC control groups (Figure 2e). As illustrated by wound-healing assay and transwell assay, circRNF20 silencing suppressed Y79 and WERI-Rb-1 cells to migrate and invade in comparison with sh-NC groups (Figure 2f and g). Moreover, the levels of proliferation-related protein (PCNA), apoptosis-related protein (cleaved-caspase 3) and epithelial–mesenchymal transition (EMT) markers (E-cadherin, N-cadherin and vimentin) in sh-circRNF20#1 transfected Y79 and WERI-Rb-1 cells were measured by western blot assay. The results showed that circRNF20 knockdown reduced the protein levels of PCNA, N-cadherin and vimentin and elevated the protein levels of cleaved-caspase 3 and E-cadherin in Y79 and WERI-Rb-1 cells (Figure 2h). Collectively, circRNF20 deficiency repressed the malignant phenotypes of RB cells.

### 3.3 CircRNF20 directly targeted miR-132-3p

**Figure 3:**
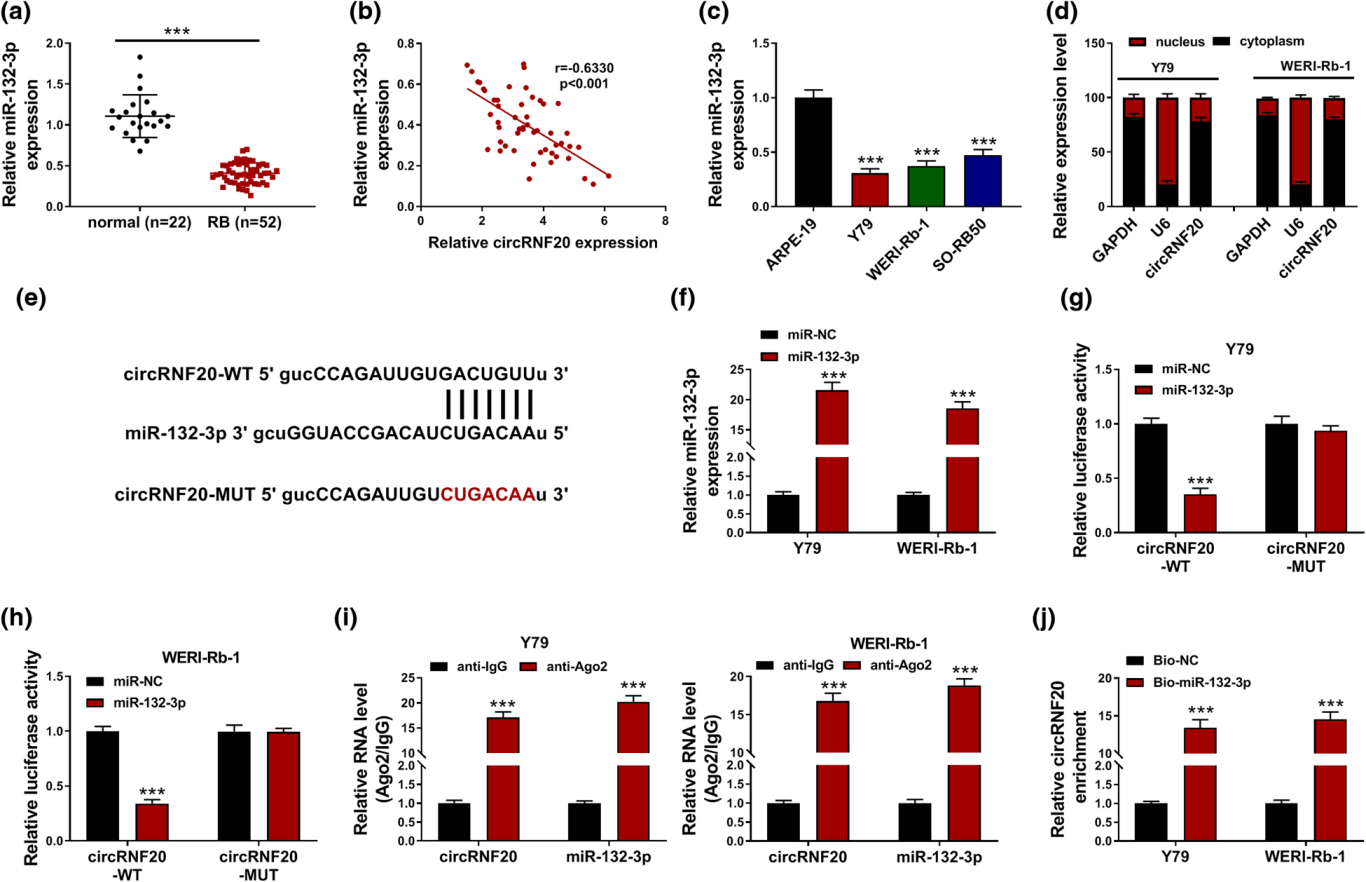
circRNF20 directly interacted with miR-132-3p. (a) The expression of miR-132-3p in RB tissues and normal tissues was detected by qRT-PCR. (b) The linear correlation between the levels of circRNF20 and miR-132-3p in RB tissues was analyzed by Spearman’s correlation coefficient analysis. (c) The expression of miR-132-3p in ARPE-19, Y79, WERI-Rb-1 and SO-RB50 was detected by qRT-PCR. (d) The distribution of circRNF20 in the cytoplasm and nucleus of Y79 and WERI-Rb-1 cells was analyzed. (e) The binding sites between circRNF20 and miR-132-3p were exhibited. (f) The expression of miR-132-3p in Y79 and WERI-Rb-1 cells with miR-132-3p or miR-NC transfection was detected by qRT-PCR assay. (g–j) The relationship between circRNF20 and miR-132-3p was investigated by dual-luciferase reporter assay, RIP assay and RNA pull-down assay. ***P < 0.001.

As shown in Figure 3a, miR-132-3p was weakly expressed in RB tissues compared to normal tissues. Of note, there was an inverse correlation between the levels of miR-132-3p and circRNF20 in RB tissues (Figure 3b). Compared to ARPE-19 cells, miR-132-3p was downregulated in Y79, WERI-Rb-1 and SO-RB50 cells (Figure 3c). Subcellular fraction analysis showed that circRNF20 was mainly enriched in the cytoplasm of Y79 and WERI-Rb-1 cells (Figure 3d). These results indicated that circRNF20 had the potential to bind to miR-132-3p. Through analyzing starbase, circRNF20 was found to contain the binding sites of miR-132-3p (Figure 3e). The mimics of miR-132-3p transfection caused a marked elevation of miR-132-3p in Y79 and WERI-Rb-1 cells (Figure 3f). Then, the relationship between circRNF20 and miR-132-3p was investigated. Dual- luciferase reporter assay showed that miR-132-3p overexpression suppressed the luciferase activity of circRNF20-WT in Y79 and WERI-Rb-1 cells, but that of circRNF20-MUT was not affected (Figure 3g and h). RIP assay showed that circRNF20 and miR-132-3p were markedly enriched in anti-Ago2 immunoprecipitated complexes compared to anti-IgG control groups (Figure 3i). RNA pull-down assay showed that Bio-miR-132-3p was able to pull down more circRNF20 in Y79 and WERI-Rb-1 cells than Bio-NC (Figure 3j). Collectively, miR-132-3p was a target of circRNF20.

### 3.4 Overexpression of miR-132-3p repressed RB cell proliferation, migration and invasion and promoted apoptosis

**Figure 4:**
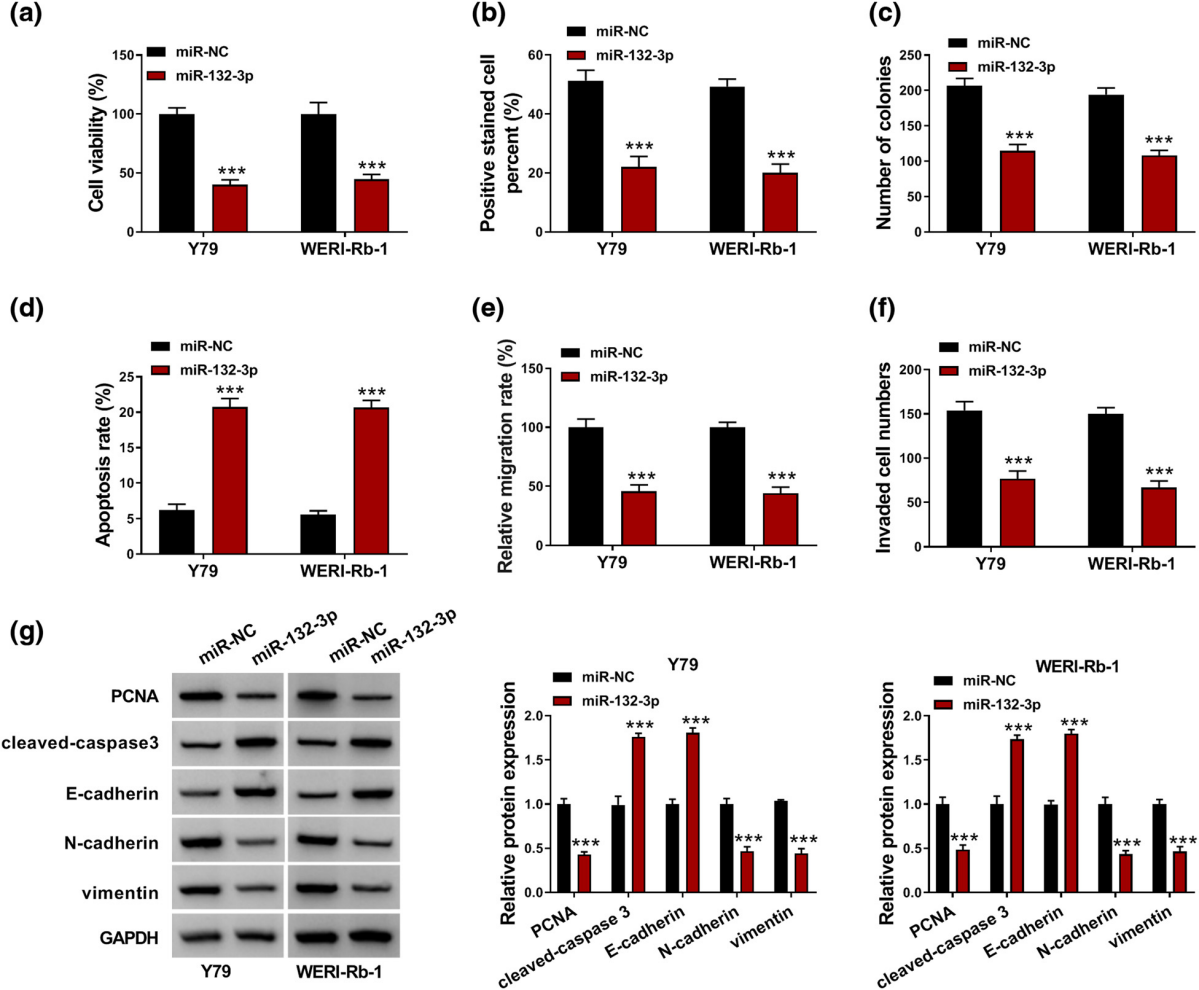
miR-132-3p overexpression inhibited RB cell progression. Y79 and WERI-Rb-1 cells were transfected with miR-NC or miR-132-3p. (a–c) The viability, proliferation and colony formation of Y79 and WERI-Rb-1 cells were evaluated by CCK-8 assay, EDU assay and colony formation assay. (d) The apoptosis of Y79 and WERI-Rb-1 cells was estimated by flow cytometry analysis. (e and f) The migration and invasion of Y79 and WERI-Rb-1 cells were assessed by wound-healing assay and transwell assay. (g) The protein levels of PCNA, cleaved-caspase 3, E-cadherin, N-cadherin and vimentin in Y79 and WERI-Rb-1 cells were measured via western blot assay. ***P < 0.001.

Next, the roles of miR-132-3p in RB development were explored. As suggested by CCK-8 assay, EDU assay and colony formation assay, miR-132-3p overexpression hampered the viability, proliferation and colony formation of Y79 and WERI-Rb-1 cells compared to miR-NC control groups (Figure 4a–c). Flow cytometry analysis indicated that the apoptosis of Y79 and WERI-Rb-1 cells was induced by increasing miR-132-3p compared to miR-NC control groups (Figure 4d). The results of wound-healing assay and transwell assay indicated that miR-132-3p elevation markedly suppressed the migration and invasion of Y79 and WERI-Rb-1 cells in comparison with miR-NC control groups (Figure 4e and f). Additionally, miR-132-3p upregulation decreased the protein levels of PCNA, N-cadherin and vimentin and increased the protein levels of cleaved-caspase 3 and E-cadherin in Y79 and WERI-Rb-1 cells (Figure 4g). Taken together, miR-132-3p played a suppressive role in RB cell progression.

### 3.5 PAX6 was the target gene of miR-132-3p

**Figure 5:**
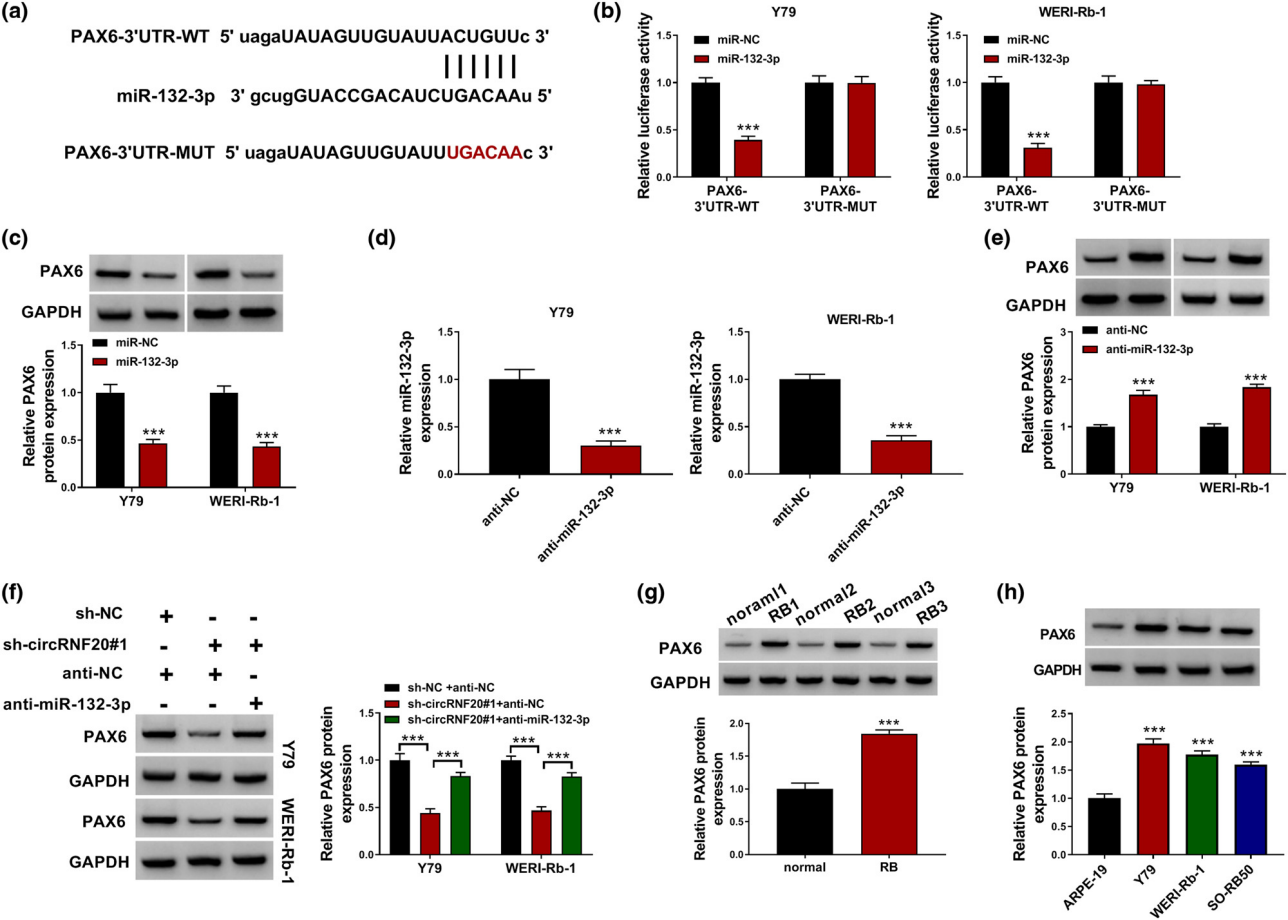
miR-132-3p directly interacted with PAX6. (a) The complementary sequences between PAX6 and miR-132-3p. (b) The interaction between miR-132-3p and PAX6 was demonstrated by dual-luciferase reporter assay. (c) The protein level of PAX6 in Y79 and WERI-Rb-1 cells transfected with miR-NC or miR-132-3p was measured by western blot assay. (d) The expression of miR-132-3p in Y79 and WERI-Rb-1 cells transfected with anti-NC or anti-miR-132-3p was determined by qRT-PCR assay. (e) The protein level of PAX7 in anti-NC or anti-miR-132-3p transfected Y79 and WERI-Rb-1 cells was measured with western blot assay. (f) The protein level of PAX6 in Y79 and WERI-Rb-1 cells with sh-NC + anti-NC, sh-circRNF20#1 + anti-NC or sh-circRNF20#1 + anti-miR-132-3p was measured through western blot assay. (g and h) The protein level of PAX6 in RB tissues and cells was examined via western blot assay. ***P < 0.001.

To analyze the potential target genes of miR-132-3p, we searched starbase and found that PAX6 was the target gene of miR-132-3p (Figure 5a). Dual-luciferase reporter assay showed that the luciferase intensity of PAX6-3′-UTR-WT was reduced by miR-132-3p overexpression, while the luciferase intensity of PAX6-3′-UTR-MUT was not changed (Figure 5b). Moreover, miR-132-3p overexpression reduced the protein level of PAX6 in Y79 and WERI-Rb-1 cells compared to miR-NC control groups (Figure 5c). As presented in Figure 5d, anti-miR-132-3p transfection markedly decreased miR-132-3p expression in Y79 and WERI-Rb-1 cells compared to anti-NC groups (Figure 5d). MiR-132-3p inhibition drastically increased PAX6 protein level in Y79 and WERI-Rb-1 cells (Figure 5e). Of note, circRNF20 knockdown decreased the PAX6 protein level in Y79 and WERI-Rb-1 cells, while miR-132-3p inhibition reversed the effect (Figure 5f). Indeed, the PAX6 protein level was upregulated by RB tissues and cells compared to normal tissues and cells (Figure 5g and h). Taken together, circRNF20 directly targeted miR-132-3p to regulate PAX6 expression.

### 3.6 miR-132-3p inhibition or PAX6 overexpression reversed the effects of circRNF20 knockdown on RB cell proliferation, apoptosis, migration and invasion

**Figure 6:**
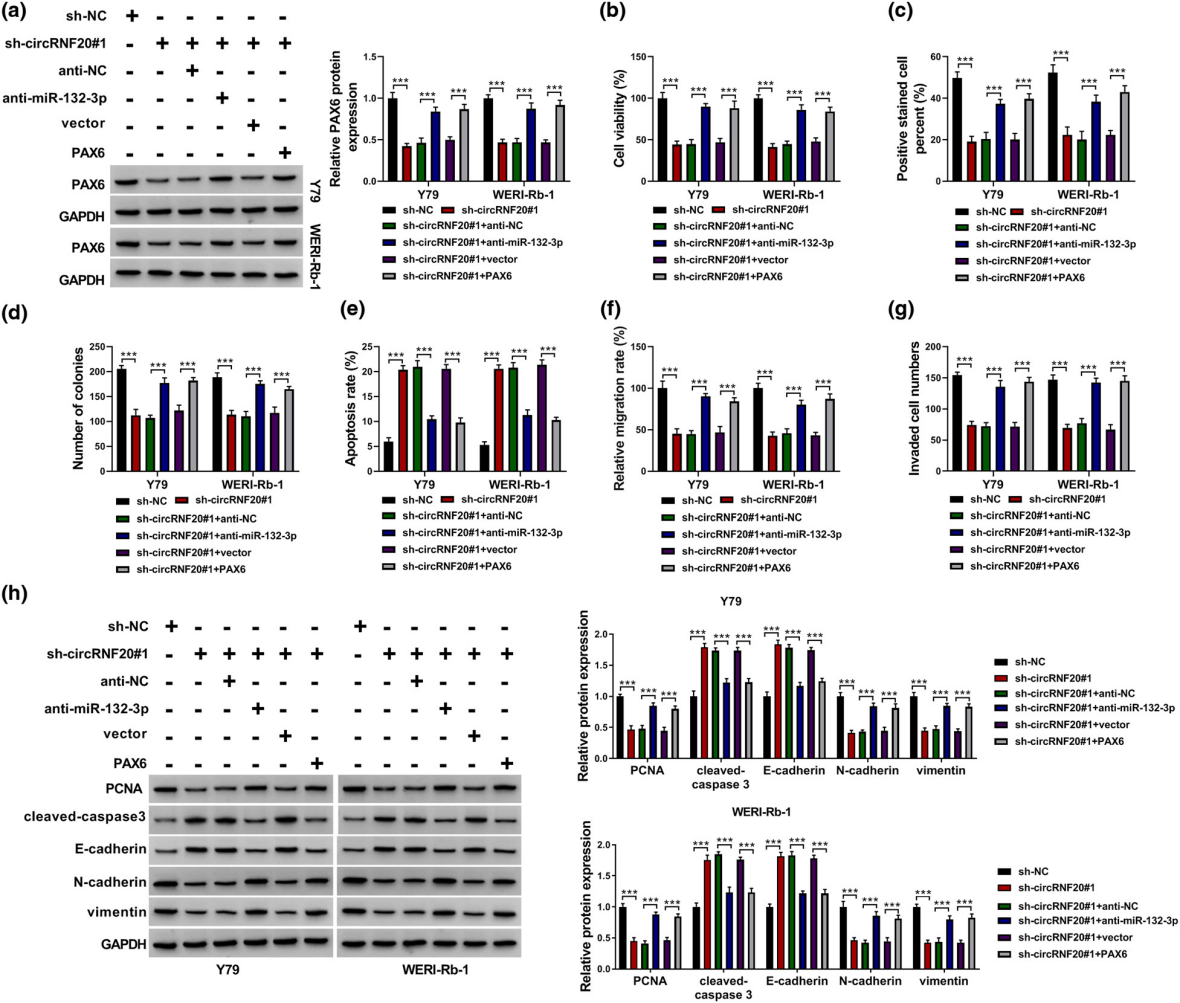
circRNF20 regulated RB cell malignant behaviors by miR-132-3p and PAX6. Sh-NC, sh-circRNF20#1, sh-circRNF20#1 + anti-NC, sh-circRNF20#1 + anti-miR-132-3p, sh-circRNF20#1 + vector or sh-circRNF20#1 + PAX6 was transfected into Y79 and WERI-Rb-1 cells. (a) The protein level of PAX6 in Y79 and WERI-Rb-1 cells was examined by qRT-PCR assay. (b–d) The viability, proliferation and colony formation of Y79 and WERI-Rb-1 cells were tested by CCK-8 assay, EDU assay and colony formation assay. (e) The apoptosis of Y79 and WERI-Rb-1 cells was analyzed by flow cytometry analysis. (f and g) The migration and invasion of Y79 and WERI-Rb-1 cells were assessed by wound-healing assay and transwell assay. (h) The protein levels of PCNA, cleaved-caspase 3, E-cadherin, N-cadherin and vimentin in Y79 and WERI-Rb-1 cells were measured by western blot assay. ***P < 0.001.

In order to clarify the relationships among circRNF20, miR-132-3p and PAX6 in RB cell progression, Y79 and WERI-Rb-1 cells were introduced with sh-NC, sh-circRNF20#1, sh-circRNF20#1 + anti-NC, sh-circRNF20#1 + anti-miR-132-3p, sh-circRNF20#1 + vector or sh-circRNF20#1 + PAX6. As exhibited in Figure 6a, circRNF20 knockdown suppressed the protein level of PAX6 in Y79 and WERI-Rb-1 cells, while miR-132-3p inhibition or PAX6 overexpression abolished the impact (Figure 6a). As suggested by CCK-8 assay, EDU assay and colony formation assay, the inhibitory functions on Y79 and WERI-Rb-1 cell viability, proliferation and colony formation mediated by circRNF20 knockdown were reversed by decreasing miR-132-3p or increasing PAX6 (Figure 6b–d). Flow cytometry analysis indicated that circRNF20 silencing facilitated the apoptosis of Y79 and WERI-Rb-1 cells, while miR-132-3p inhibition or PAX6 overexpression abated the effect (Figure 6e). The suppressive roles of circRNF20 knockdown in Y79 and WERI-Rb-1 cell migration and invasion were also rescued following miR-132-3p inhibition or PAX6 elevation (Figure 6f and g). Additionally, miR-132-3p downregulation or PAX6 upregulation reversed the effects of circRNF20 silencing on the protein levels of PCNA, cleaved-caspase 3, E-cadherin, N-cadherin and vimentin in Y79 and WERI-Rb-1 cells (Figure 6h). Collectively, circRNF20 knockdown impeded RB cell progression via miR-132-3p/PAX6 axis.

### 3.7 circRNF20 knockdown restrained tumor formation *in vivo*

**Figure 7:**
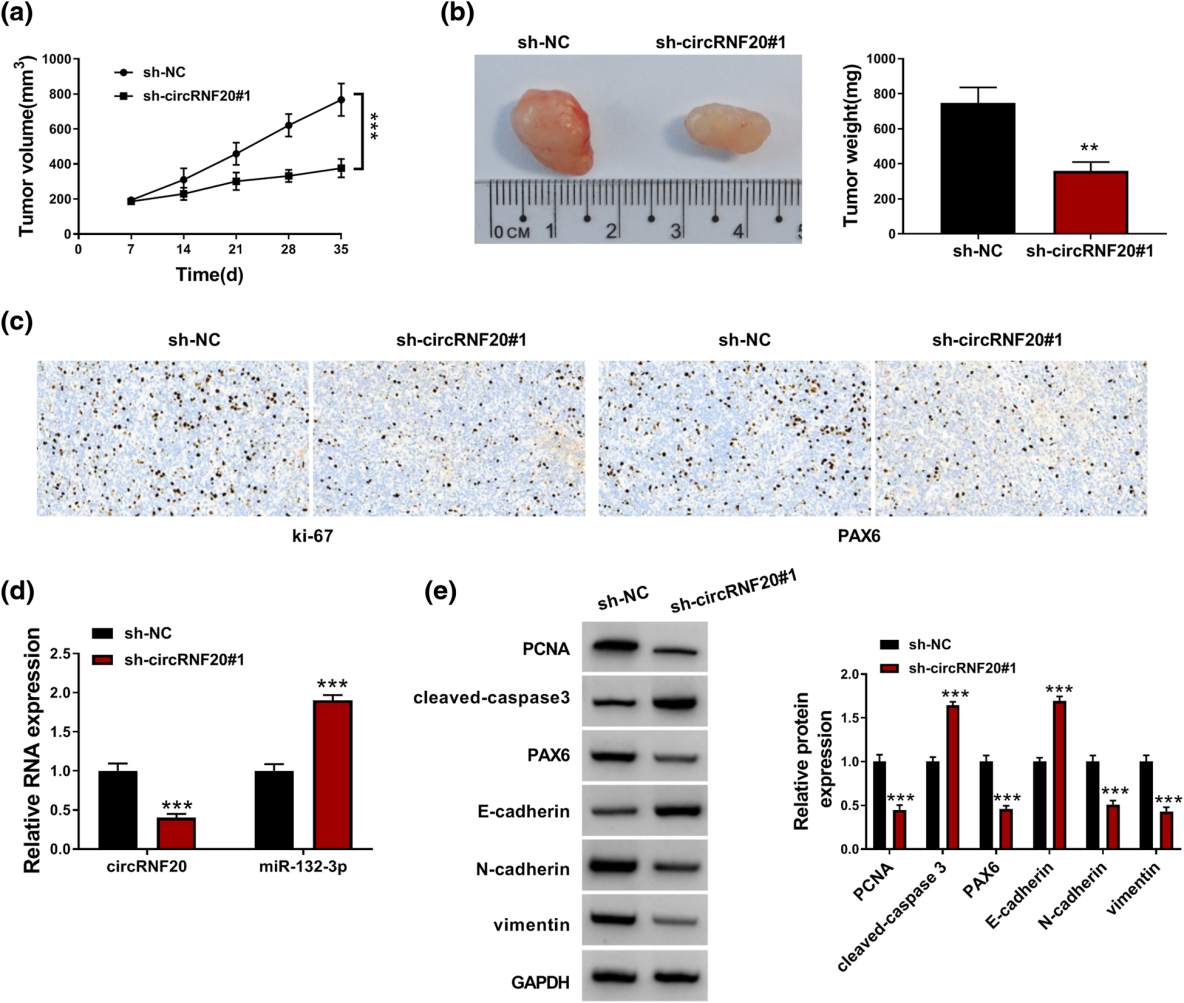
circRNF20 silencing blocked tumor growth *in vivo*. (a and b) The volume and weight of xenograft tumor were examined. (c) The levels of ki-67 and PAX6 in xenograft tumors were examined by IHC assay. (d) The levels of circRNF20 and miR-132-3p in xenograft tumors were detected by qRT-PCR assay. (e) The protein levels of PCNA, cleaved-caspase 3, PAX6, E-cadherin, N-cadherin and vimentin in xenograft tumors were measured via western blot assay. **P < 0. 01, ***P < 0.001.

Afterward, the role of circRNF20 in tumor growth *in vivo* was investigated. As a result, circRNF20 silencing restrained tumor growth (including tumor volume and tumor weight) (Figure 7a and b). IHC assay showed that ki-67 and PAX6 levels were reduced in the xenograft tumors in sh-circRNF20#1 groups compared to sh-NC groups (Figure 7c). The tumors in sh-circRNF20#1 groups also exhibited reduced circRNF20 expression and elevated miR-132-3p expression relative to sh-NC groups (Figure 7d). In addition, the protein levels of PCNA, PAX6, N-cadherin and vimentin were decreased and the protein levels of cleaved-caspase 3 and E-cadherin were increased in the xenograft tumors in sh-circRNF20#1 groups compared to sh-NC control groups (Figure 7e). All these results suggested that circRNF20 played an antitumor effect on RB progression.

### 3.8 Exosomal circRNF20 promoted RB cell growth, migration and invasion and repressed apoptosis

**Figure 8:**
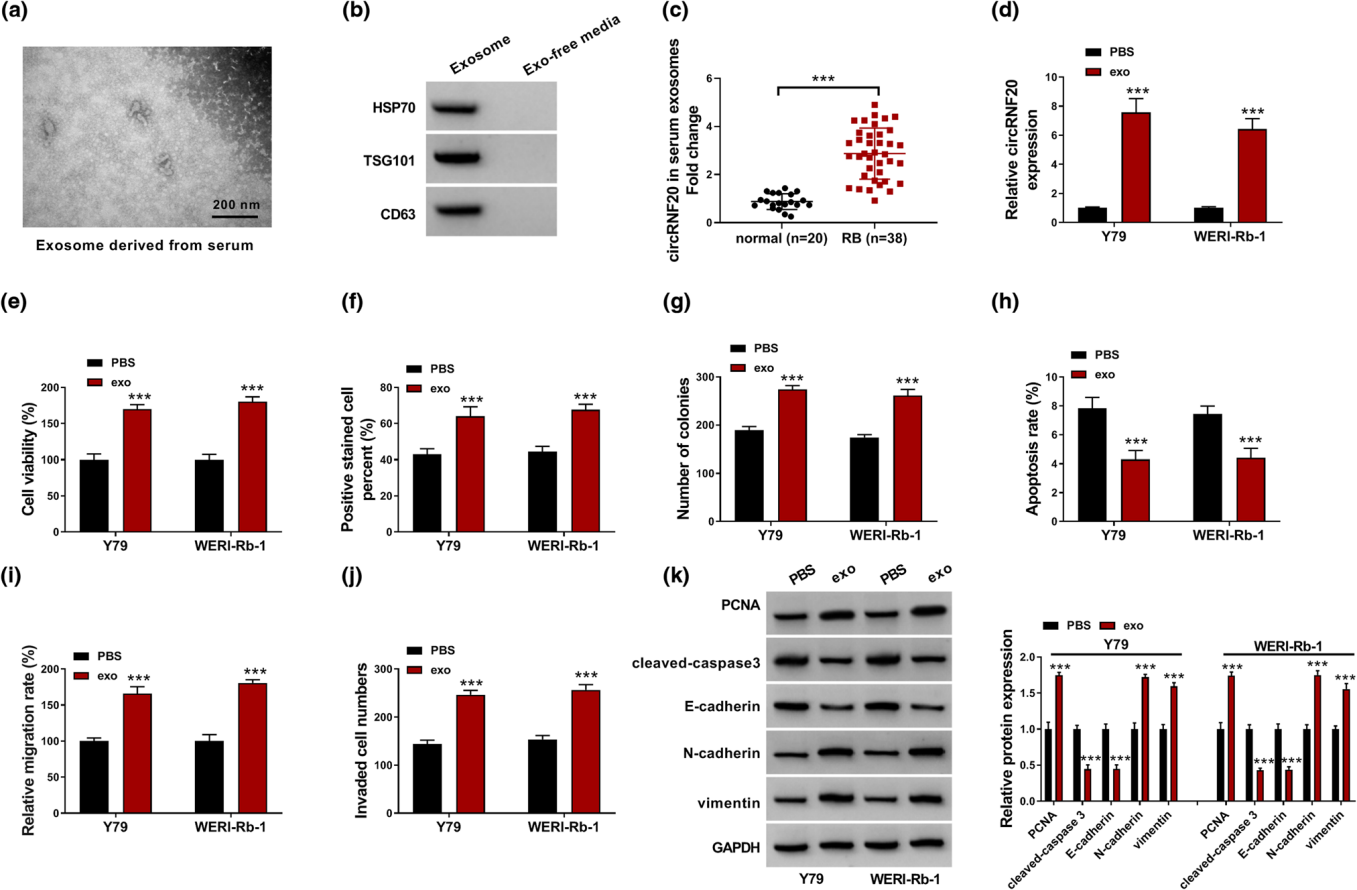
Effects of circRNF20 derived from RB patients’ serums on RB cell growth, apoptosis and metastasis. (a) The morphology of exosomes was analyzed by TEM. (b) The protein levels of HSP70 and TSG101 were measured by western blot assay. (c) The level of circRNF20 in the exosomes derived from the serums of RB patients and normal controls was detected by qRT-PCR assay. (d) The expression of circRNF20 in Y79 and WERI-Rb-1 cells incubated with RB patients serums’ isolated exosomes was detected by qRT-PCR assay. (e–j) After Y79 and WERI-Rb-1 cells were incubated with the exosomes derived from RB patient serums, cell viability, proliferation, apoptosis, migration and invasion were analyzed. (k) The protein levels of PCNA, cleaved-caspase 3, E-cadherin, N-cadherin and vimentin were measured through western blot assay. ***P < 0.001.

At last, the exosomes were isolated from the serums of RB patients and the morphology is presented in Figure 8a. The exosomal markers HSP70 and TSG101 could be detected in the obtained exosomes (Figure 8b). qRT-PCR assay showed that circRNF20 was upregulated in the exosomes derived from RB patients’ serums (Figure 8c). Then, the exosomes were incubated with Y79 and WERI-Rb-1 cells and then the circRNF20 expression level was detected. The results exhibited that the circRNF20 level was increased in Y79 and WERI-Rb-1 cells incubated with RB patients’ serum-derived exosomes (Figure 8d). Moreover, exosome incubation promoted the viability, proliferation and colony formation of Y79 and WERI-Rb-1 cells (Figure 8e–g). Flow cytometry analysis showed that exosome incubation repressed the apoptosis of Y79 and WERI-Rb-1 cells (Figure 8h). The results of wound-healing assay and transwell assay indicated that the migration and invasion of Y79 and WERI-Rb-1 cells were inhibited after exosome incubation (Figure 8i and j). In addition, PCNA, N-cadherin and vimentin levels were increased and cleaved-caspase 3 and E-cadherin levels were decreased in Y79 and WERI-Rb-1 cells incubated with exosomes (Figure 8k). These results indicated that exosomal circRNF20 might promote RB cell progression.

## 4 Discussion

Currently, with the development of bioinformatics technology and sequencing technology, RB-related circRNAs have been gradually discovered [25,26]. Even so, limited circRNAs are reported to be linked to RB. Herein, we aimed to clarify the functions of circRNF20 in RB malignancy. It was found that circRNF20 served as an oncogene in RB through miR-132-3p/PAX6 axis.

Cao et al. reported that the raised circRNF20 level was related to poor clinical outcome in breast cancer and accelerated cancer progression by the miR-487a/HIF-1α/HK2 pathway [14]. Wang et al. unraveled that circRNF20 triggered the proliferation of NSCLC [13]. These reports suggested that circRNF20 played a vital role in human cancer development. In this work, the abnormal elevation of circRNF20 was discovered in RB. circRNF20 elevation was linked to advanced TNM stage and worse overall survival rate. Rapid growth and metastasis are the characteristics of RB [27]. Herein, our results exhibited that circRNF20 silencing restrained RB cell growth, migration and invasion and triggered apoptosis. EMT refers to epithelial to mesenchymal cell transformation, which gives cells the ability to migrate and invade [28]. Thus, in this study, we determined the expression of EMT-related markers. It was found that circRNF20 deficiency reduced E-cadherin level and increased N-cadherin and vimentin levels in RB cells, indicating the repression of EMT process. Besides, the tumor-suppressive effect of circRNF20 silencing was demonstrated via *in vivo* experiments.

Afterward, miR-132-3p was demonstrated to be the target of circRNF20 and circRNF20 negatively regulated miR-132-3p expression by sponging miR-132-3p. Previous studies showed that miR-132-3p could be targeted by circ-sirt1 or circ_DOCK1 to influence tumor progression [29,30]. Nevertheless, the relationship between circRNF20 and miR-132-3p was first verified. Moreover, miR-132-3p elevation restrained RB cell growth and invasion [18]. In the present research, miR-132-3p overexpression curbed cell proliferation and motility and stimulated apoptosis in RB cells. Remarkably, miR-132-3p suppression could abrogate circRNF20 silencing-mediated impacts on RB cell malignant phenotypes. In addition, PAX6 was identified as the target for miR-132-3p. Previous studies showed that PAX6 aggravated RB cell growth, migration and invasion and inhibited apoptosis [20,21,22]. In this study, PAX6 enhancement rescued the impact of circRNF20 silencing on RB cell progression, indicating the promotional effect of circRNF20 on RB development.

Exosomes are extracellular vesicles with 40–100 nm, which can be secreted by almost all cells [31,32]. Exosomes exert physiological roles in cell-to-cell communication by secreting proteins, DNA, lipids, circRNAs, miRNAs, mRNAs and other biomolecules [31]. Exosomal circRNAs can regulate the biological process of tumor cells and function as important biomarkers in human cancers [33,34,35]. In this study, our results presented that circRNF20 was upregulated in RB patients’ serum-derived exosomes. Moreover, exosomal circRNF20 boosted cell proliferation and metastasis and repressed apoptosis in RB cells. These results suggested that exosomal circRNF20 promoted RB progression and might be a biomarker for RB diagnosis.

Taken together, circRNF20 aggravated the progression of RB by influencing miR-132-3p/PAX6 axis. These findings might offer a promising strategy for RB therapy. Moreover, exosomal circRNF20 level in serums might be a diagnostic marker for RB.

## References

[1] Dimaras H, Kimani K, Dimba EA, Gronsdahl P, White A, Chan HS, et al. Retinoblastoma. Lancet. 2012;379(9824):1436–46. 10.1016/S0140-6736(11)61137-9 22414599

[2] AlAli A, Kletke S, Gallie B, Lam WC. Retinoblastoma for Pediatric ophthalmologists. Asia Pac J Ophthalmol (Phila). 2018;7(3):160–8. 10.22608/APO.201870 29737052

[3] Yun J, Li Y, Xu CT, Pan BR. Epidemiology and Rb1 gene of retinoblastoma. Int J Ophthalmol. 2011;4(1):103–9. 10.3980/j.issn.2222-3959.2011.01.24 PMC3340672 22553621

[4] Dimaras H, Dimba EA, Gallie BL. Challenging the global retinoblastoma survival disparity through a collaborative research effort. Br J Ophthalmol. 2010;94(11):1415–6. 10.1136/bjo.2009.174136 20679076

[5] Balmer A, Zografos L, Munier F. Diagnosis and current management of retinoblastoma. Oncogene. 2006;25(38):5341–9. 10.1038/sj.onc.1209622 16936756

[6] Chen LL, Yang L. Regulation of circRNA biogenesis. RNA Biol. 2015;12(4):381–8. 10.1080/15476286.2015.1020271 PMC4615371 25746834

[7] Zhong Y, Du Y, Yang X, Mo Y, Fan C, Xiong F, et al. Circular RNAs function as ceRNAs to regulate and control human cancer progression. Mol Cancer. 2018;17(1):79. 10.1186/s12943-018-0827-8 PMC5889847 29626935

[8] Sui C, Qu W, Lian Y, Feng C, Zhan Y. Hsa_circ_0069094 knockdown inhibits cell proliferation, migration, invasion and glycolysis, while induces cell apoptosis by miR-661/HMGA1 axis in breast cancer. Anticancer Drugs. 2021;32:829–41. 10.1097/CAD.0000000000001076 33929992

[9] Wang J, Luo J, Wu X, Gao Z. Circular RNA_0000629 Suppresses Bladder Cancer Progression Mediating MicroRNA-1290/CDC73. Cancer Manag Res. 2021;13:2701–15. 10.2147/CMAR.S292863 PMC7997432 33790645

[10] Fu C, Wang S, Jin L, Zhang M, Li M. CircTET1 Inhibits Retinoblastoma Progression via Targeting miR-492 and miR-494-3p through Wnt/beta-catenin Signaling Pathway. Curr Eye Res. 2021;46(7):978–87. 10.1080/02713683.2020.1843685 33108919

[11] Jiang Y, Xiao F, Wang L, Wang T, Chen L. Circular RNA has_circ_0000034 accelerates retinoblastoma advancement through the miR-361-3p/ADAM19 axis. Mol Cell Biochem. 2021;476(1):69–80. 10.1007/s11010-020-03886-5 32844346

[12] Xing L, Zhang L, Feng Y, Cui Z, Ding L. Downregulation of circular RNA hsa_circ_0001649 indicates poor prognosis for retinoblastoma and regulates cell proliferation and apoptosis via AKT/mTOR signaling pathway. Biomed Pharmacother. 2018;105:326–33. 10.1016/j.biopha.2018.05.141 29864621

[13] Wang ZX, Zhao Y, Wang YB, Zhang Q, Zou QX, Liang FH, et al. circRNF20 aggravates the progression of non-small-cell lung carcinoma by activating MAPK9. Eur Rev Med Pharmacol Sci. 2020;24(19):9981–9. 10.26355/eurrev_202010_23211 33090403

[14] Cao L, Wang M, Dong Y, Xu B, Chen J, Ding Y, et al. Circular RNA circRNF20 promotes breast cancer tumorigenesis and Warburg effect through miR-487a/HIF-1alpha/HK2. Cell Death Dis. 2020;11(2):145. 10.1038/s41419-020-2336-0 PMC7039970 32094325

[15] Lu TX, Rothenberg ME. MicroRNA. J Allergy Clin Immunol. 2018;141(4):1202–7. 10.1016/j.jaci.2017.08.034 PMC5889965 29074454

[16] Li L, Yu H, Ren Q. MiR-218-5p Suppresses the Progression of Retinoblastoma Through Targeting NACC1 and Inhibiting the AKT/mTOR Signaling Pathway. Cancer Manag Res. 2020;12:6959–67. 10.2147/CMAR.S246142 PMC7418178 32821163

[17] Zhang C, Wu S. microRNA -378a-3p Restrains the Proliferation of Retinoblastoma Cells but Promotes Apoptosis of Retinoblastoma Cells via Inhibition of FOXG1. Invest Ophthalmol Vis Sci. 2020;61(5):31. 10.1167/iovs.61.5.31 PMC7405766 32428232

[18] Han S, Song L, Chen Y, Hou M, Wei X, Fan D. The long non-coding RNA ILF3-AS1 increases the proliferation and invasion of retinoblastoma through the miR-132-3p/SMAD2 axis. Exp Cell Res. 2020;393(2):112087. 10.1016/j.yexcr.2020.112087 32407730

[19] Jordan T, Hanson I, Zaletayev D, Hodgson S, Prosser J, Seawright A, et al. The human PAX6 gene is mutated in two patients with aniridia. Nat Genet. 1992;1(5):328–32. 10.1038/ng0892-328 1302030

[20] Meng B, Wang Y, Li B. Suppression of PAX6 promotes cell proliferation and inhibits apoptosis in human retinoblastoma cells. Int J Mol Med. 2014;34(2):399–408. 10.3892/ijmm.2014.1812 PMC4094585 24939714

[21] Li X, Yang L, Shuai T, Piao T, Wang R. MiR-433 inhibits retinoblastoma malignancy by suppressing Notch1 and PAX6 expression. Biomed Pharmacother. 2016;82:247–55. 10.1016/j.biopha.2016.05.003 27470361

[22] Li J, You X. MicroRNA758 inhibits malignant progression of retinoblastoma by directly targeting PAX6. Oncol Rep. 2018;40(3):1777–86. 10.3892/or.2018.6563 30015924

[23] Wang J, Wang X, Wu G, Hou D, Hu Q. MiR-365b-3p, down-regulated in retinoblastoma, regulates cell cycle progression and apoptosis of human retinoblastoma cells by targeting PAX6. FEBS Lett. 2013;587(12):1779–86. 10.1016/j.febslet.2013.04.029 23660406

[24] Wang L, Shang X, Feng Q. LncRNA TATDN1 contributes to the cisplatin resistance of non-small cell lung cancer through TATDN1/miR-451/TRIM66 axis. Cancer Biol Ther. 2019;20(3):261–71. 10.1080/15384047.2018.1529091 PMC6370371 30481109

[25] Guo N, Liu XF, Pant OP, Zhou DD, Hao JL, Lu CW. Circular RNAs: novel promising biomarkers in ocular diseases. Int J Med Sci. 2019;16(4):513–8. 10.7150/ijms.29750 PMC6535655 31171902

[26] Lyu J, Wang Y, Zheng Q, Hua P, Zhu X, Li J, et al. Reduction of circular RNA expression associated with human retinoblastoma. Exp Eye Res. 2019;184:278–85. 10.1016/j.exer.2019.03.017 30917906

[27] Narang S, Mashayekhi A, Rudich D, Shields CL. Predictors of long-term visual outcome after chemoreduction for management of intraocular retinoblastoma. Clin Exp Ophthalmol. 2012;40(7):736–42. 10.1111/j.1442-9071.2012.02757.x 22300311

[28] Dongre A, Weinberg RA. New insights into the mechanisms of epithelial-mesenchymal transition and implications for cancer. Nat Rev Mol Cell Biol. 2019;20(2):69–84. 10.1038/s41580-018-0080-4 30459476

[29] Li QK, Liu YK, Li JW, Liu YT, Li YF, Li BH. Circ-sirt1 inhibits growth and invasion of gastric cancer by sponging miR-132-3p/miR-212-3p and upregulating sirt1 expression. Neoplasma. 2021;68:780–7. 10.4149/neo_2021_210218N222 34034499

[30] Zhang W, Wang Z, Cai G, Huang P. Circ_DOCK1 regulates USP11 through miR-132-3p to control colorectal cancer progression. World J Surg Oncol. 2021;19(1):67. 10.1186/s12957-021-02173-x PMC7941900 33685455

[31] Azmi AS, Bao B, Sarkar FH. Exosomes in cancer development, metastasis, and drug resistance: a comprehensive review. Cancer Metastasis Rev. 2013;32(3–4):623–42. 10.1007/s10555-013-9441-9 PMC3843988 23709120

[32] Thery C, Zitvogel L, Amigorena S. Exosomes: composition, biogenesis and function. Nat Rev Immunol. 2002;2(8):569–79. 10.1038/nri855 12154376

[33] Xie M, Yu T, Jing X, Ma L, Fan Y, Yang F, et al. Exosomal circSHKBP1 promotes gastric cancer progression via regulating the miR-582-3p/HUR/VEGF axis and suppressing HSP90 degradation. Mol Cancer. 2020;19(1):112. 10.1186/s12943-020-01208-3 PMC7322843 32600329

[34] Wu G, Zhou W, Pan X, Sun Z, Sun Y, Xu H, et al. Circular RNA profiling reveals exosomal circ_0006156 as a novel biomarker in papillary thyroid cancer. Mol Ther Nucleic Acids. 2020;19:1134–44. 10.1016/j.omtn.2019.12.025 PMC7016027 32059339

[35] Liu D, Kang H, Gao M, Jin L, Zhang F, Chen D, et al. Exosome-transmitted circ_MMP2 promotes hepatocellular carcinoma metastasis by upregulating MMP2. Mol Oncol. 2020;14(6):1365–80. 10.1002/1878-0261.12637 PMC7266270 31944556

